# Enhanced Function and Overexpression of Metabotropic Glutamate Receptors 1 and 5 in the Spinal Cord of the SOD1^G93A^ Mouse Model of Amyotrophic Lateral Sclerosis during Disease Progression

**DOI:** 10.3390/ijms20184552

**Published:** 2019-09-13

**Authors:** Tiziana Bonifacino, Claudia Rebosio, Francesca Provenzano, Carola Torazza, Matilde Balbi, Marco Milanese, Luca Raiteri, Cesare Usai, Ernesto Fedele, Giambattista Bonanno

**Affiliations:** 1 Department of Pharmacy, Unit of Pharmacology and Toxicology, University of Genoa, 16148 Genova, Italy; bonifacino@difar.unige.it (T.B.); claudiarebosio@gmail.com (C.R.); provenzano.phd@difar.unige.it (F.P.); torazza@difar.unige.it (C.T.); balbi.phd@difar.unige.it (M.B.); 2 Department of Pharmacy, Unit of Pharmacology and Toxicology and Center of Excellence for Biomedical Research (CEBR), University of Genoa, 16132 Genova, Italy; milanese@difar.unige.it (M.M.); lraiter@difar.unige.it (L.R.); fedele@difar.unige.it (E.F.); 3 Institute of Biophysics, National Research Council (CNR), 16149 Genova, Italy; usai@ge.ibf.cnr.it; 4 Istituto di Ricovero e Cura a Carattere Scientifico (IRCCS) Ospedale Policlinico San Martino, 16132 Genova, Italy

**Keywords:** amyotrophic lateral sclerosis, SOD1^G93A^ mice, spinal cord, metabotropic glutamate receptor 1 (mGluR1), metabotropic glutamate receptor 5 (mGluR5), glutamate release, mGluR1/mGluR5 expression, disease progression

## Abstract

Glutamate (Glu)-mediated excitotoxicity is a major cause of amyotrophic lateral sclerosis (ALS) and our previous work highlighted that abnormal Glu release may represent a leading mechanism for excessive synaptic Glu. We demonstrated that group I metabotropic Glu receptors (mGluR1, mGluR5) produced abnormal Glu release in SOD1^G93A^ mouse spinal cord at a late disease stage (120 days). Here, we studied this phenomenon in pre-symptomatic (30 and 60 days) and early-symptomatic (90 days) SOD1^G93A^ mice. The mGluR1/5 agonist (*S*)-3,5-Dihydroxyphenylglycine (3,5-DHPG) concentration dependently stimulated the release of [^3^H]d-Aspartate ([^3^H]d-Asp), which was comparable in 30- and 60-day-old wild type mice and SOD1^G93A^ mice. At variance, [^3^H]d-Asp release was significantly augmented in 90-day-old SOD1^G93A^ mice and both mGluR1 and mGluR5 were involved. The 3,5-DHPG-induced [^3^H]d-Asp release was exocytotic, being of vesicular origin and mediated by intra-terminal Ca^2+^ release. mGluR1 and mGluR5 expression was increased in Glu spinal cord axon terminals of 90-day-old SOD1^G93A^ mice, but not in the whole axon terminal population. Interestingly, mGluR1 and mGluR5 were significantly augmented in total spinal cord tissue already at 60 days. Thus, function and expression of group I mGluRs are enhanced in the early-symptomatic SOD1^G93A^ mouse spinal cord, possibly participating in excessive Glu transmission and supporting their implication in ALS. Please define all abbreviations the first time they appear in the abstract, the main text, and the first figure or table caption.

## 1. Introduction

Amyotrophic lateral sclerosis (ALS), originally described by Charcot in 1869, is a fatal progressive neurodegenerative disease characterized by damage and death of upper and lower motor neurons (MNs), leading to muscle wasting, weakness, and spasticity [1]. Although primary symptoms of ALS are associated with motor dysfunction, up to 50% of patients develop cognitive and/or behavioral impairment during the course of the disease and 13% present frontotemporal dementia [2,3,4]. The incidence of the disease is approximately 3 cases per 100,000 individuals per year, with a male:female ratio of almost 2 [5]. To date, no drug is available to effectively counteract ALS progression. Only two drugs have been approved for therapy: riluzole, which improves survival and quality of life only modestly [6], and, very recently, edaravone, which shows some efficacy only in patients at the early stage of the disease [7].

ALS can be sporadic (sALS), representing more than 90% of cases, or familial (fALS), which is genetically transmissible and accounts for about 10% of cases. More than 30 genes involved in fALS have been identified [8,9] and four of them, chromosome 9 open reading frame 72 (*C9ORF72*), superoxide dismutase 1 (*SOD1*), TAR DNA-binding protein 43 (*TARDBP*), and mutations in fused in sarcoma protein (*FUS*), are responsible for more than 70% of cases [10]. The pathogenesis of ALS is still unknown, although clinical symptoms and disease progression are similar in sALS and fALS, indicating that common mechanisms may be involved. Vulnerability of MNs has been ascribed to numerous causes, including protein misfolding, mitochondrial dysfunction, oxidative damage, defective axonal transport, glutamate-mediated excitotoxicity, insufficient growth factor signaling, and inflammation [11,12,13,14]. Moreover, the damage of MNs is enhanced by damage occurring in non-neuronal neighboring cells, such as astrocytes and microglia, thus accelerating disease progression [15,16,17].

High levels of extracellular glutamate (Glu) have been detected in an elevated percentage of sALS and fALS patients [18,19,20], suggesting that excitotoxicity plays an important role in motor neuron degeneration. Impaired glutamate clearance, due to a reduced expression of the astrocyte Glu transporters, has been proposed as the cause triggering excessive synaptic Glu [21,22,23,24]. However, Glu transport defects do not seem to be the only origin of excessive extracellular Glu, since it has been shown that the spontaneous release of Glu and that induced by depolarization [25], by group I metabotropic Glu receptors (mGluRs) [26] or GABA and glycine hetero-transporter [27,28,29] activation, is abnormal in the mouse model of human ALS expressing a high copy number of mutant human superoxide dismutase 1 (SOD1) with a Gly93Ala substitution (SOD1^G93A^) [30].

In particular, we have demonstrated that group I mGluRs (including mGluR1 and mGluR5) produced abnormal Glu release in the spinal cord of 120-day-old SOD1^G93A^ mice, which represent the late stage of the disease. Concentrations of the mGluR1/5 agonist (*S*)-3,5-Dihydroxyphenylglycine (3,5-DHPG) > 0.3 μM stimulated the release of Glu to the same extent in the control and SOD1^G93A^ mice. At variance, concentrations ≤ 0.3 μM of 3,5-DHPG increased Glu release in SOD1^G93A^ mice only. Both mGluR1 and mGluR5 were involved, with mGluR5 being preferentially responsible for the high potency effects of 3,5-DHPG. The release of Glu induced by 3,5-DHPG was exocytotic in nature and it was sustained by intra-terminal Ca^2+^ release through inositol 1,4,5-triphosphate (IP3)-sensitive Ca^2+^ channels.

Interestingly, the abnormal Glu release induced by hetero-transporter activation or axon terminal membrane depolarization in SOD1^G93A^ mice was also detectable before symptoms occur, as early as 30 days of life [31,32], suggesting that it can represent a cause rather than a consequence of disease progression. This precociousness has not been explored in the case of Glu release induced by activation of group I mGluRs. Therefore, in the present work, we investigated the modulation of Glu release by mGluR1 and mGluR5 and the expression of these receptors in the SOD1^G93A^ spinal cord at the pre-symptomatic (30 and 60 days) and early-symptomatic (90 days) stages of the disease.

## 2. Results

### 2.1. Effects of 3,5-DHPG on [^3^H]d-Aspartate Release during Disease Progression

**Figure 1:**
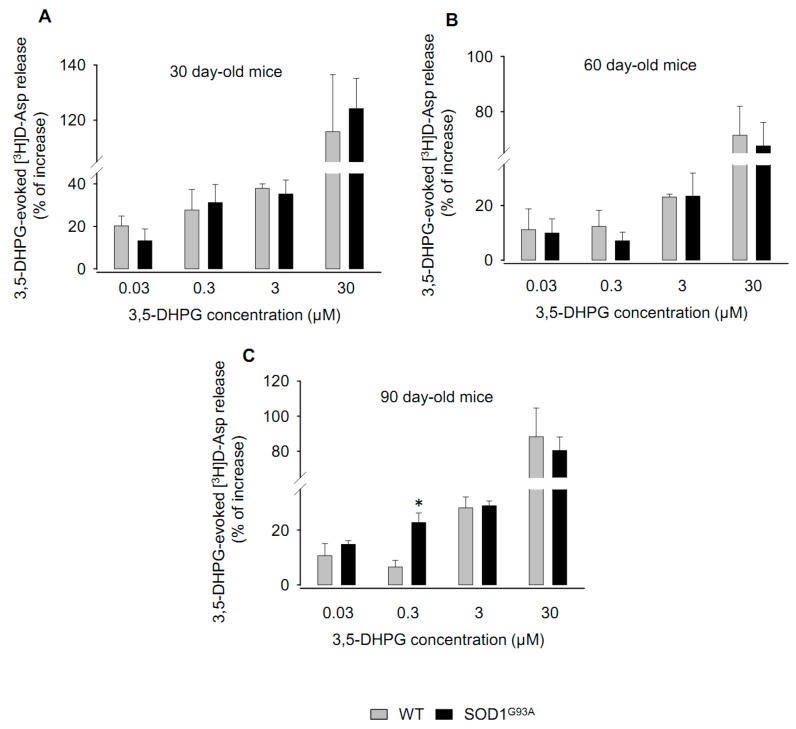
Release of [^3^H]d-Aspartate ([^3^H]d-Asp) in spinal cord synaptosomes purified from wild type mice (WT) and SOD1^G93A^ mice. The effect of (*S*)-3,5-dihydroxyphenylglycine (3,5-DHPG) on the spontaneous release of [^3^H]d-Asp was measured in spinal cord synaptosomes prepared from SOD1^G93A^ mice at different stages of the disease: (**A**) 30 days of life, (**B**) 60 days of life, and (**C**) 90 days of life, corresponding to pre-symptomatic (30 and 60 days) and early-symptomatic (90 days) phases of the disease. Age-matched WT animals were used as controls. Synaptosomes were loaded with [^3^H]d-Asp, in order to label the intra-terminal releasing pools of Glu, and exposed in superfusion to increasing concentrations (0.03, 0.3, 3, and 30 µM) of 3,5-DHPG. Labelling by [^3^H]d-Asp did not differ significantly between WT and SOD1^G93A^ mice when normalized for synaptosomal protein. Superfusion samples collected were counted for radioactivity. Results are expressed as the percent increase respect to the basal control release. The data reported are the means ± SEM of five independent experiments (five WT and five SOD1^G93A^ mice) run in triplicate (three superfusion chambers for each experimental condition). No significance was detected in 30- and 60-day-old mice, while a significant difference was found in 90-day-old SOD1^G93A^ mice versus WT mice. * *p* < 0.001 vs. WT (two-way ANOVA followed by Bonferroni’s post-hoc test).

Here, we studied the release of [^3^H]d-Aspartate ([^3^H]d-Asp) induced by the mixed mGluR1/5 agonist 3,5-DHPG [33] from 30-, 60-, and 90-day-old WT and SOD1^G93A^ mouse synaptosomes in superfusion. The amount of tritium loaded into the preparation per mg of protein did not vary under the different conditions tested. The exposure of spinal cord synaptosomes to 3,5-DHPG (0.03–30 µM) concentrations dependently stimulated the release of [^3^H]d-Asp in wild type mice (WT) and SOD1^G93A^ mice at the three disease stages (Figure 1A–C). No differences were observed between WT and SOD1^G93A^ mice at 30 (Figure 1A; *F*_(1,3,3,33)_ = 0.189) or 60 (Figure 1B; *F*_(1,3,3,17)_ = 0.0423) days of age. Also, the exposure of 90-day-old spinal cord synaptosomes to increasing concentrations of 3,5-DHPG generated similar effects in WT and SOD1^G93A^ mice, except at 0.3 µM 3,5-DHPG, which produced a significant increase of the release in SOD1^G93A^ with respect to WT mice (Figure 1C; *p* < 0.05; *F*_(1,3,3,48)_ = 1.502).

These data show that the 3,5-DHPG-induced [^3^H]d-Asp release is abnormally increased in 90-day-old SOD1^G93A^ mice, but not at earlier disease stages.

### 2.2. Mechanisms Underlying the Modulation of [^3^H]d-Aspartate Release by 3,5-DHPG in 90-Day-Old Mice

In order to define the mGlu receptor subtype involved in the abnormal [^3^H]d-Asp release induced by 0.3 µM 3,5-DHPG in 90-day-old SOD1^G93A^ mice, we tested the competitive mGluR1 antagonist (*S*)-(+)-a-amino-4-carboxy-2-methylbenzeneacetic acid (LY367385) [34] and the non-competitive mGluR5 antagonist 2-methyl-6-(phenylethynyl)pyridine (MPEP) [35] on the 3,5-DHPG-releasing effect.

**Figure 2:**
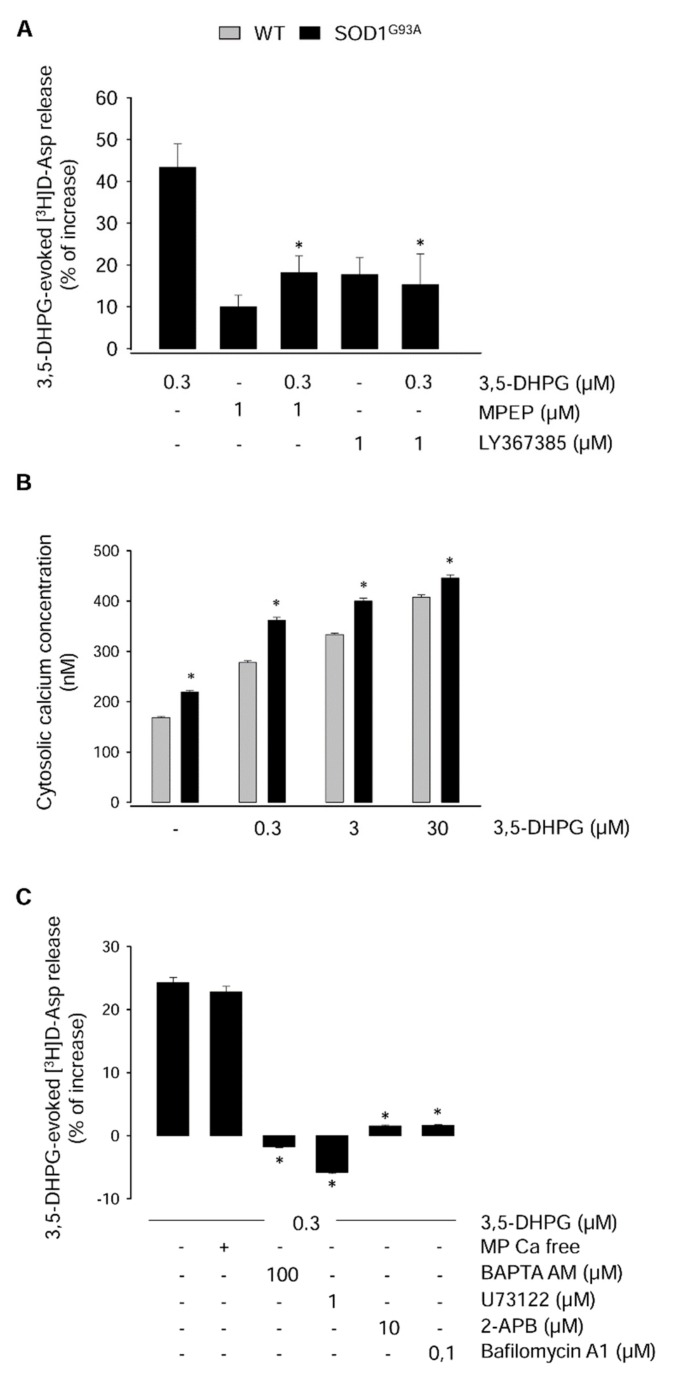
Mechanisms underlying the 3,5-DHPG-evoked [^3^H]d-Asp release in spinal cord synaptosomes from 90-day-old WT and SOD1^G93A^ mice. (**A**) The effects of mGluR1 and mGluR5 selective antagonists were tested on the 0.3 µM 3,5-DHPG-evoked [^3^H]d-Asp release. Spinal cord synaptosomes were exposed in superfusion to 1 µM LY367385 or MPEP, 8 min before and concomitantly with 0.3 µM 3,5-DHPG. See the legend of Figure 1 for other technical details. The data reported are the means ± SEM of five independent experiments (five WT and five SOD1^G93A^ mice) run in triplicate (three superfusion chambers for each experimental condition). * *p* < 0.05 vs. 3,5-DHPG (one-way ANOVA followed by Bonferroni’s post-hoc test). (**B**) Resting and 3,5-DHPG-evoked [Ca^2+^]_C_ were measured in spinal cord synaptosomes purified from 90-day-old SOD1^G93A^ and age-matched WT control mice. Synaptosomes were labelled with fura-2-acetoxymethyl ester (fura 2-AM) and exposed to standard medium or to 0.3, 3, and 30 µM 3,5-DHPG. [Ca^2+^]_C_ was determined fluorometrically as described in the Methods section. Data are means ± SEM of three independent experiments (three WT and three SOD1^G93A^ mice) run in triplicate (three experimental replicates). * *p* < 0.001 vs. WT mice (two-way ANOVA followed by Bonferroni’s post-hoc test). (**C**) The effects of external calcium absence (Ca-free medium), cytosolic calcium chelation by 1,2-bis(2-aminophenoxy)ethane-*N*,*N*,*N*′,*N*′-tetraacetic acid tetrakis acetoxymethyl ester (BAPTA-AM) (100 µM), phospholipase C inhibition by 1-[6-[((17β)-3-methoxyestra-1,3,5[10]-trien-17-yl)amino]hexyl]-1H-pyrrole-2,5-dione (U73122) (1 µM), IP3 receptor blockade by 2-aminoethoxydiphenyl borate (2-APB) (10 µM), or vesicular neurotransmitter deprivation by bafilomycin A1 (0.1 µM) were tested on the 0.3 µM 3,5-DHPG-evoked [^3^H]d-Asp release in spinal cord synaptosomes purified from 90-day-old SOD1^G93A^ and age-matched WT mice. Spinal cord synaptosomes were labelled with [^3^H]d-Asp and exposed in superfusion to 0.3 µM 3,5-DHPG and to the above mentioned experimental conditions, as described under the Methods section. The presence of BAPTA-AM or bafilomycin A1 did not significantly affect synaptosomal labelling by [^3^H]d-Asp. Results are expressed as the percent increase with respect to the basal control release. The data reported are the means ± SEM of four independent experiments (four WT and four SOD1^G93A^ mice) run in triplicate (three superfusion chambers for each experimental condition). * *p* < 0.001 vs. the effect of 3,5-DHPG (one-way ANOVA followed by Dunnett’s post-hoc test).

The 0.3 µM 3,5-DHPG-induced [^3^H]d-Asp release was strongly reduced by both antagonists, used at the concentration of 1 µM (Figure 2A; *p* < 0.05; *F*_(4,9)_ = 8.422), thus indicating that both mGluR1 and mGluR5 contribute to the abnormal release of [^3^H]d-Asp observed at this disease stage.

Since mGluR1 and mGluR5 induce Ca^2+^ mobilization form intracellular stores [36], we studied the cytosolic calcium concentration ([Ca^2+^]_C_) in spinal cord synaptosomes from 90-day-old WT and SOD1^G93A^ mice, under basal conditions and following exposure to 3,5-DHPG by labelling with the fura-2-acetoxymethyl ester (FURA 2-AM) fluorescent dye. As shown in Figure 2B, the [Ca^2+^]_C_ in the absence of 3,5-DHPG was significantly more elevated in synaptosomes from SOD1^G93A^ with respect to WT mice (*p* < 0.001; *F*_(1,3,3,30)_ = 11.154). Exposure to 0.3, 3, or 30 µM 3,5-DHPG induced a concentration-dependent increase of [Ca^2+^]_C_ in both mouse strains (*p* < 0.001; *F*_(1,3,3,30)_ = 11.154). 3,5-DHPG produced a further [Ca^2+^]_C_ increase, leading to a total [Ca^2+^]_C_ that was significantly greater in SOD1^G93A^ mice than that measured in WT mice (*p* < 0.001; *F*_(1,3,3,30)_ = 11.154).

In order to verify the origin of Ca^2+^ producing the increase of [Ca^2+^]_C_, we analyzed the release of [^3^H]d-Asp in spinal cord synaptosomes from 90-day-old SOD1^G93A^ mice exposed to 0.3 µM 3,5-DHPG in the absence of external Ca^2+^, as well as in Ca^2+^-containing standard medium but in synaptosomes incubated in the presence of 100 µM 1,2-bis(2-aminophenoxy)ethane-*N*,*N*,*N*′,*N*′-tetraacetic acid tetrakis acetoxymethyl ester (BAPTA-AM) prior to release experiments, to block intracellular Ca^2+^ [37], or exposed in superfusion to the phospholipase C inhibitor 1-[6-[((17β)-3-methoxyestra-1,3,5[10]-trien-17-yl)amino]hexyl]-1H-pyrrole-2,5-dione (U73122) (1 µM) [38] or to the IP3 receptor blocker 2-aminoethoxydiphenyl borate (2-APB) (10 µM) [39]. Figure 2C shows that omission of Ca^2+^ in the superfusion medium did not reduce the release of [^3^H]d-Asp evoked by 3,5-DHPG.

On the contrary, the effect of 3,5-DHPG on [^3^H]d-Asp release was abolished by BAPTA-AM in the presence of external Ca^2+^, by 2-APB (*p* < 0.001; *F*_(5,28)_ = 567.116), or by U73122 (*p* < 0.001; *F*_(5,28)_ = 567.116). Figure 2C also shows that the effect of 0.3 µM 3,5-DHPG was occluded by pre-incubation of synaptosomes with 0.1 µM bafilomycin A1, a drug that depletes the vesicular neurotransmitter content [40] (*p* < 0.001; *F*_(5,28)_ = 567.116).

These data demonstrate that [Ca^2+^]_C_ is abnormally augmented in spinal cord synaptosomes from 90-day-old SOD1^G93A^ mice because of its release from endoplasmic reticulum stores through IP3 receptor channels and that the increase of the release of [^3^H]d-Asp by 0.3 µM 3,5-DHPG is exocytotic in nature.

### 2.3. Expression of mGluR1 and mGluR5 in Spinal Cord Glutamatergic Synaptosomes during Disease Progression

**Figure 3:**
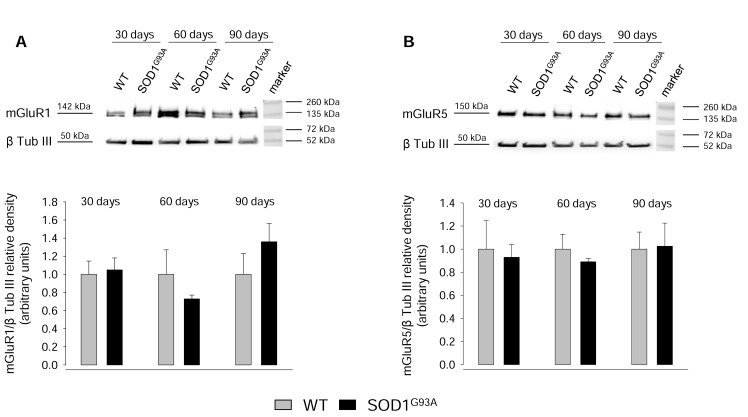
mGluR1 and mGluR5 expression in spinal cord synaptosomes from WT and SOD1^G93A^ mice. SOD1^G93A^ mice were sacrificed at postnatal day 30, 60, and 90 days. Age-matched WT mice were used as controls. (**A**) mGluR1 and (**B**) mGluR5 expression were measured by SDS 4–20% polyacrylamide gel electrophoresis (PAGE) and western blot (WB) onto nitrocellulose membranes, using 20 µg of detergent soluble protein extracts from the homogenate of spinal cord synaptosomes. Mouse monoclonal antibodies anti-mGlu1 receptor, rabbit monoclonal antibody anti-mGluR5, and mouse monoclonal antibody anti-β-tubulin III were used. Representative immunoreactive bands are shown. mGlu1 and mGlu5 receptor expression levels were normalized for β-tubulin III in the same blotted membrane and the quantization is reported in the bar plots. Results are expressed as the relative density and the expression of mGluR1 or mGluR5 in WT synaptosomes is referred to as 1.00. Means ± SEM of three independent experiments (three WT and three SOD1^G93A^ mice) are presented. No significant differences were detected (two-tailed student’s *t*-test).

In order to evaluate possible changes in the expression of mGluR1 and mGluR5 during disease progression, we carried out western blot (WB) experiments using protein extracts of spinal cord synaptosomes from 30-, 60-, 90-day-old WT and SOD1^G93A^ mice. Figure 3A shows that mGluR1 expression did not change significantly in SOD1^G93A^ mice with respect to age-matched WT mice at any age tested. Similar results were obtained with regard to mGluR5 expression (Figure 3B).

Since total synaptosomal lysates may include heterogeneous populations of spinal cord synaptic terminals differently expressing the two metabotropic receptors, we carried out immunofluorescence studies to investigate the levels of mGluR1 and GluR5 selectively expressed at glutamatergic axon terminals during disease progression.

**Figure 4:**
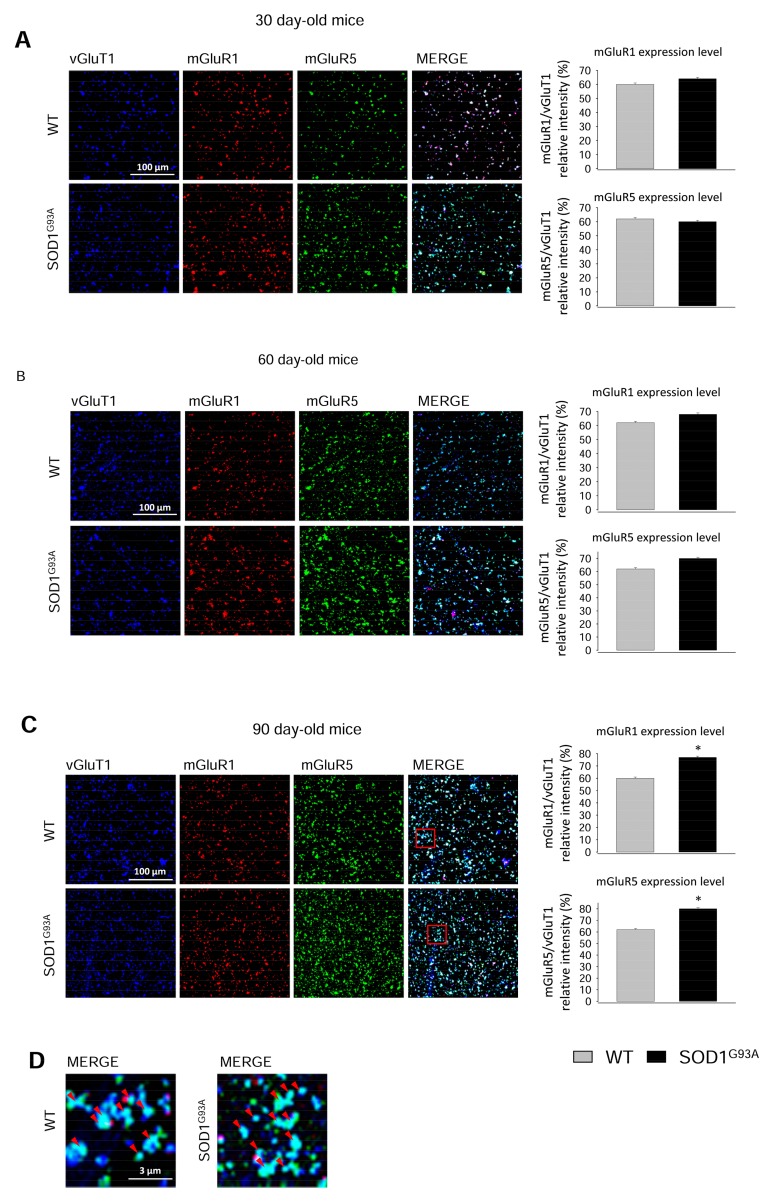
mGluR1 and mGluR5 expression in glutamatergic spinal cord synaptosomes from WT and SOD1^G93A^ mice. SOD1^G93A^ mice were sacrificed at different stages of the disease: (**A**) 30 days of life, (**B**) 60 days of life, and (**C**) 90 days of life, corresponding to pre-symptomatic (30 and 60 days) and early-symptomatic (90 days) phases of the disease. Age-matched WT mice were used as controls. Spinal cord synaptosomes were layered onto coverslips, fixed with paraformaldehyde, permeabilized with triton X-100, and incubated with the specific primary and secondary antibodies, as described in the Methods section. Samples were analyzed by laser confocal microscopy. The acquired images show the triple-stained immune-positivity for Alexa Fluor 488-tagged anti-vGluT1 (blue; glutamatergic synaptosomes), Alexa Fluor 647-tagged anti-mGluR1 (red), and Alexa Fluor 555-tagged anti-mGluR5 (green). The merge panels show the co-expression of vGluT1, mGluR1, and mGluR5. Bar plots indicate the quantitative relative florescence intensity of mGluR1 or mGluR5 in vGluT1-positive spinal cord synaptosomes. Representative images are shown (scale bar = 100 µm). mGluR1 and mGluR5 expression levels in vGluT1-positive synaptosomes were calculated. Means ± SEM of three independent experiments (three WT and three SOD1^G93A^ mice) run in triplicate (three experimental replicates) are presented. No significant differences were detectable in spinal cord glutamatergic synaptosomes from 30- and 60-day-old WT and SOD1^G93A^ mice. On the contrary, the expression of mGluR1 and mGluR5 was significantly higher in synaptosomes purified from 90-day-old SOD1^G93A^ mice. * *p* < 0.001 vs. WT mice (two-tailed student’s *t*-test). (**D**) shows a magnification of the red frames in merge panels (reported in Figure 4C) of spinal cord synaptosomes purified from 90-day-old WT and SOD1^G93A^ mice. Red arrows indicate axon terminals triple stained for vGluT1, mGluR1, and mGluR5 (scale bar = 3 µm).

Confocal microscopy experiments were performed by staining spinal cord synaptosomes for the vesicular Glu transporter 1 (vGluT1), to label glutamatergic axon terminals, and for mGluR1 or mGluR5 (Figure 4). Synaptosomal preparations efficiently stained for all the antibodies tested, thus allowing the optimal analysis of the relative fluorescence intensity of mGluR1 or mGluR5 co-localizing with vGluT1. The expression of vGluT1 did not vary between WT and SOD1^G93A^ mice and this allowed its use to calculate the relative intensity of mGluR1 or mGluR5 in glutamatergic synaptosomes. Figure 4A, B shows that the relative intensity of mGluR1 and mGluR5 was not significantly modified at 30 and 60 days, although a trend toward an increase can be observed at the latter stage of the disease. On the contrary, both mGluR1 and mGluR5 were significantly increased in SOD1^G93A^ mice at 90 days of life compared to WT mice (Figure 4C; mGluR1, *p* < 0.001, *t*_(4)_ = −12.021; mGluR5, *p* < 0.001, *t*_(4)_ = −12.728).

Figure 4D report a magnification of the merge panels of Figure 4C (red frames), obtained using spinal cord synaptosomes purified from 90-day-old WT and SOD1^G93A^ mice and shows that a large number of vGluT1-positive glutamatergic synaptosomes also co-express mGluR1 and mGluR5 (red arrows).

Therefore, mGluR1 and mGluR5 were over-expressed at glutamatergic axon terminals in 90-day-old SOD1^G93A^ mice.

### 2.4. Expression of mGluR1 and mGluR5 in Spinal Cord Total Tissue during Disease Progression

Group I mGluRs are present in neurons at the pre- and post-synaptic level, as well as in non-neuronal cells, such as astrocytes, microglia and oligodendrocytes [41,42,43].

**Figure 5:**
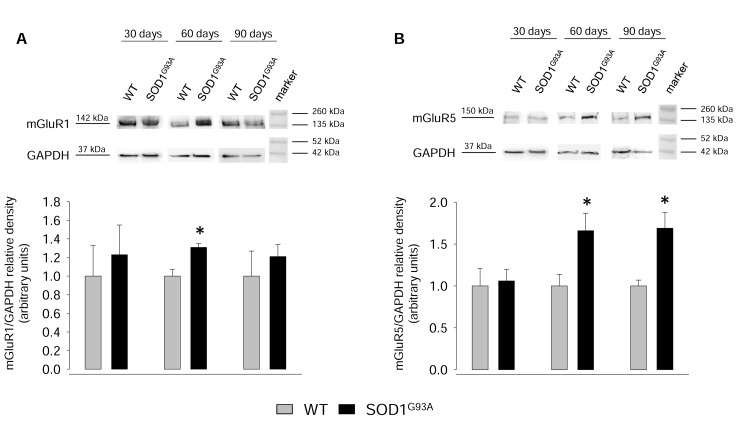
mGluR1 and mGluR5 expression in spinal cord total tissue from WT and SOD1^G93A^ mice. SOD1^G93A^ mice were sacrificed at postnatal day 30, 60, and 90. Age-matched WT mice were used as controls. (**A**) mGluR1 and (**B**) mGluR5 expression were measured by SDS 4–20% PAGE and WB onto nitrocellulose membranes, using 20 µg of detergent soluble protein extracts from the homogenate of the whole spinal cord. Mouse monoclonal antibody anti-mGluR1, rabbit monoclonal antibody anti-mGluR5, and mouse monoclonal antibody anti-glyceraldehyde 3-phosphate dehydrogenase (GAPDH) were used. mGluR1 and mGluR5 expression levels were normalized for GAPDH in the same membrane. Representative immunoreactive bands and quantization (bar plots) are shown. mGluR1 and mGluR5 levels are expressed as the relative density of SOD1^G93A^ with respect to WT mouse bands, which is referred to as 1.00. Bars report mean ± SEM of five independent experiments (five WT and five SOD1^G93A^ mice) run in triplicate (three experimental replicates) are presented. No significant differences were detected in 30-day-old mice, while significant differences were found in 60- and 90-day-old mice. * *p* < 0.05 vs. WT mice (two-tailed student’s *t*-test).

The data reported in the previous paragraph suggest that mGluR1 and mGluR5 expression does not change significantly in the total synaptosomal population of SOD1^G93A^ mice, being upregulated, however, at the glutamatergic axon terminals. In order to ascertain whether these receptors are also overexpressed in SOD1^G93A^ mice at non-presynaptic sites (i.e., post-synaptically and/or in non-neuronal cells), we conducted WB experiments on protein extracted from the whole spinal cord tissue of 30-, 60-, and 90-day-old WT and SOD1^G93A^ mice. The results obtained overall show that group I mGluRs are overexpressed during the progression of the pathology compared to WT mice, with mGluR1 expression significantly increased at 60 days (Figure 5A; *p* < 0.05, *t*_(4)_ = −3.148) and mGluR5 expression significantly augmented in SOD1^G93A^ mice at 60 and 90 days of life (Figure 5B; *p* < 0.05, *t*_(8)_ = −2.615 and *p* < 0.05, *t*_(6)_ = −3.374, respectively).

These data suggest that mGluR1 and mGluR5 are overexpressed in the spinal cord of SOD1^G93A^ mice during disease progression even at early stages, and that this overexpression can be attributed mainly to non-presynaptic structures.

## 3. Discussion

Group I metabotropic glutamate receptors (mGluR1 and mGluR5) are actively involved in the regulation of important cellular processes altered in ALS and play a key role in the complex ALS scenario [44,45,46,47,48,49,50]. Starting from our previous results showing that mGluR1 and mGluR5 abnormally increase the release of glutamate in the spinal cord of late symptomatic SOD1^G93A^ mice [26], we here investigated the expression and function of these receptors during disease progression to unveil whether they can also modify glutamate transmission at more precocious stages of the disease.

Our functional results show that 3,5-DHPG induced Glu release in WT and SOD1^G93A^ mice at 30, 60, and 90 days of life. Comparing the releasing effects observed in this work with previous results in 120-day-old mice [26], it appears that the mGluR1/5 agonist exhibited the same efficacy (maximal effect about 100% of potentiation at 30 µM), but lower potency, since the potentiation observed at concentrations below 30 µM were always lower at 30, 60, and 90 days than at 120 days. In this work, 3,5-DHPG effects were comparable in WT and SOD1^G93A^ mice at 30 and 60 days at any concentration tested. On the contrary, 0.3 µM 3,5-DHPG was able to evoke a significant increase of Glu release (approximately 25%) in 90-day-old SOD1^G93A^ mice whereas it was ineffective in age-matched WT mice. This difference may be consistent with that observed previously at 120 days since in that case, as in the present experiments, the effects of 10 and 30 µM 3,5-DHPG were comparable in WT and SOD1^G93A^ mice and differences were observed at 1 µM and below, with 0.3 µM the highest concentration of 3,5-DHPG producing Glu release in SOD1^G93A^, but not in WT mice. On the basis of the previous results, we tested 0.03, 0.3, 3, and 30 µM 3,5-DHPG and, possibly due to the lower 3,5-DHPG potency observed here, concentrations below 0.3 µM 3,5-DHPG were unable to disclose any further difference. Alternatively, this may indicate that a sort of time-dependent iper-activation of mGluR1 and mGluR5 autoreceptors develops during the progression of the disease.

Our experiments with selective antagonists highlighted that both receptors are involved in the Glu release potentiation seen at 90 days, since either the mGluR1 antagonist LY367385 or the mGluR5 antagonist MPEP virtually abolished the release of glutamate induced by 3,5-DHPG, suggesting the existence of a functional interplay between the two receptors, as already proposed in late-symptomatic SOD1^G93A^ mice [26]. The data from confocal microscopy experiments, indicating the coexistence of mGluR1 and mGluR5 on the same vGluT1-positive synaptosomes, support the results of release experiments. Interestingly, mGlu1 and mGlu5 receptor–receptor interaction has been proposed after measuring Glu release in mouse cerebral cortex synaptosomes by the same experimental set-up used here [51], and the existence of mGluR heterodimers, including mGluR1/mGluR5, has been directly demonstrated in different cell lines [52]. Thus, functional heterodimers might exist also in spinal cord synaptosomes and we would attempt to speculate that the different expression of mGluR1 and mGluR5 in 90-day-old SOD1^G93A^ mice could lead to the assembly of active heterodimers with different stoichiometry, supporting a modified function, and thus explaining why 3,5-DHPG was able to stimulate Glu release with a high affinity in SOD1^G93A^ mice only.

The excessive 3,5-DHPG-induced Glu release was of vesicular origin, being abolished by bafilomycin A1, which depletes the vesicle protonic gradient, hampering the storing of neurotransmitters [40]. Additionally, it is exocytotic in nature since it was triggered by an increase of [Ca^2+^]_C_ and reduced by cytosolic Ca^2+^ buffering by BAPTA-AM, suggesting that Ca^2+^ originates by intra-terminal stores. Moreover, the results obtained with the PLC inhibitor, U73122 [38], and the IP3 channel blocker, 2-APB [39], demonstrate that the effect of 3,5-DHPG on Glu release is mediated by classical metabotropic receptors belonging to the group I mGluRs coupled to membrane phospholipid hydrolysis, IP3 formation, and Ca^2+^ mobilization from the endoplasmic reticulum. We also found a dysregulation of the synaptic Ca^2+^ homeostasis at the axon terminal level, characterized by resting overload and increased levels also after 3,5-DHPG exposure. Whether 3,5-DHPG can augment by itself the [Ca^2+^]_C_ more efficiently in SOD1^G93A^ mice is difficult to say on the basis of the present experiments, and the extent of [Ca^2+^]_C_ increase produced by the agonist would suggest that the basal level plays a pivotal role. Whatever the case may be, the control of Ca^2+^ homeostasis is altered in SOD1^G93A^ mice, leading to increased [Ca^2+^]_C_. If we assume that a minimal threshold of [Ca^2+^]_C_ is needed to trigger Glu release, this condition would be more easily obtained in SOD1^G93A^ mice that show a higher basal level. Moreover, increased [Ca^2+^]_C_ can activate various Ca^2+^-dependent signaling cascades involved in presynaptic function and exocytosis processes, including the calpain-calpastatin protease system, that indeed we found profoundly altered in SOD1^G93^ mice [53,54] and that could, in turn, also affect release mechanisms [55].

Interestingly, abnormal exocytotic Glu release in the SOD1^G93A^ mouse spinal cord does not seem to be a unique characteristic of mGluRs since it was also observed using high KCl, ionomycin, or hypertonic sucrose as releasing stimuli [25]. These stimuli exploit different mechanisms to produce Ca^2+^-dependent and Ca^2+^-independent neurotransmitter release and it was determined that their excessive effects are based on plastic changes of the Glu axon terminal biochemistry, leading to increased phosphorylation of synapsin I and other key release-regulating proteins, increased number of membrane-bound core complexes, and increased size of the readily releasable pool of vesicles [25,32]. It will be of interest to determine in future studies whether mGluR1 and mGluR5 adopt the same mechanisms to produce the augmented Glu release in SOD1^G93A^ mice. Furthermore, at variance with the previously reported abnormal exocytotic release of Glu induced by depolarization of axon terminals, which was detectable as early as at 30 days of life in SOD1^G93A^ mice [32], the present release modifications take place between 60 and 90 days. As a consequence, we can assume that the modification of the autoreceptor function at Glu synaptic terminals represents a consequence of disease progression rather than a cause of the disease itself.

The release of glutamate was studied using purified synaptosomes stratified as a thin layer on a support and up-down superfused with physiological medium in the presence or in the absence of 3,5-DHPG and of the other tested substances. According to the characteristics of this original technique [56,57], the mGluR1 and mGluR5 that modulate the release of Glu are localized onto Glu-releasing axon terminals in the spinal cord. In line with these functional data, confocal microscopy experiments in the mouse spinal cord revealed an increased expression of both mGluR1 and mGluR5 in vGluT1-stained (i.e., glutamatergic) synaptosomes obtained from early symptomatic 90-day-old SOD1^G93A^ mice, when compared to WT mice. Differently, glutamatergic axon terminals derived from the spinal cord of pre-symptomatic 30- or 60-day-old SOD1^G93A^ mice proved only a non-significant tendency to the increase of mGluR1 or mGluR5 expression, suggesting that the abnormal activity of Glu release-regulating group I mGluRs parallels the development of disease symptoms. Interestingly, no changes in the expression of the two receptors were detected in the spinal cord synaptosomal homogenate prepared from 30-, 60-, and 90-day-old SOD1^G93A^ mice, indicating that the presynaptic overexpression of mGluR1 and mGluR5 preferentially or exclusively affects the glutamatergic subpopulation of spinal cord axon terminals. At variance, an increase of mGluR1 and mGluR5 expression was previously observed in WB experiments using synaptosomal homogenates obtained from 120-day-old SOD1^G93A^ [26], suggesting that, at this disease stage, the overexpression at glutamatergic axon terminals is even more pronounced and/or that other synaptic terminals could be involved.

Noteworthy, an overexpression of mGluR1 and mGluR5 was registered in the total spinal cord tissue, but not in synaptosomes, between 30 and 60 days of life in SOD1^G93A^ mice, suggesting that other non-presynaptic receptors may play a role at earlier stages. As to the possible anatomical location of these up-regulated non-presynaptic receptors, they might be located in spine and dendritic arborizations at the postsynaptic level, where they modulate Glu transmission and multiple related events [58], as well as in non-neuronal cells, such as astrocytes, microglia, and oligodendrocytes [59], which is attractive in view of the well-known non-cell autonomous characteristics of the disease [16]. In fact, it is now clear that astrocytes [17,60] and microglia [15,61] play a crucial role in MNs degeneration during disease progression. Recent studies have shown that oligodendrocytes are also involved in the non-cell autonomous nature of ALS [62,63]. Disclosing the role played by mGluR1 and mGluR5 in the different non neuronal cell populations would be of crucial importance for a better understanding of the neurodegenerative processes taking place in ALS and for the development of effective pharmacological approaches.

The abnormal activity of mGluR1 and mGluR5 demonstrated previously [26] and in the present work further support the rationale for the therapeutic exploitation of reducing mGluR1/5 activity. Indeed, we demonstrated that the genetic reduction [64,65] or ablation [66] of mGluR1 or mGluR5 in SOD1^G93A^ mice positively affects the disease course in SOD1^G93A^ mice, prolonging survival, ameliorating motor skills, and improving biochemical and cellular parameters that are altered during the progression of ALS. These in vivo data are supported by previous in vitro evidence indicating positive effects of group I mGluR activity inhibition on MNs degeneration [67,68,69]. The in vivo beneficial effects of down regulating group I mGluR expression in ALS indicate that the amelioration of the disease progression may additionally be due to down-regulation of receptors other than those expressed on Glu-releasing axon terminals. The present demonstration that in total spinal cord tissue, mGluR1 and mGluR5 are over expressed even earlier than those sited at Glu axon terminals supports the above idea. Thus, the group I mGluRs genetic down regulation should create a virtuous cycle, reducing both the number of receptors and their stimulation by released Glu.

To recapitulate, we demonstrated for the first time that the mGluR1- and mGluR5-releasing function is altered in SOD1^G93A^ mice starting from an early-symptomatic stage of the disease and that these receptors are overexpressed in the spinal cord even at pre-symptomatic disease stages. This could have implication for ALS in vivo. It has been shown that chronic treatment with the prototypic mGluR5 antagonist MPEP attenuates cell death in vitro and delays the onset of motor symptoms and slightly prolongs survival in vivo in SOD1^G93A^ mice [49]. Selective and more potent mGluR5 antagonists, with more favorable pharmacokinetics, are now available and have been tested in preclinical studies in rodents [70,71,72,73,74,75] and in clinical trials in patients [76,77,78] for pathologies different from ALS, showing a favorable safety profile and good efficacy. A positive outcome of in vivo pharmacological studies, in ALS mouse models, with mGluR5 antagonists, already tested in patients, would pave the way to rapid translational application to human ALS. Neuroprotective polytherapy with receptor antagonists aimed at blocking both mGluR1 and mGluR5 could be even more efficacious.

In conclusion, group I mGluRs promise to be helpful druggable targets for pharmacological interventions in ALS and further preclinical single- or multi-drug therapies in animal models of the disease could obtain encouraging results, paving the way for clinical interventions in patients.

## 4. Materials and Methods

### 4.1. Animals

Adult B6SJL-TgN SOD1/G93A(þ)1Gur mice expressing a high copy number of mutant human SOD1 with a Gly93Ala substitution [SOD1^G93A^] [30] were originally obtained from Jackson Laboratories (Bar Harbor, ME, USA). They were bred at the animal facility of the Pharmacology and Toxicology Unit of the Department of Pharmacy at the University of Genoa. Transgenic animals were crossed with background-matched B6SJL wild-type female, and selective breeding maintained each transgene in the hemizygous state. Non-transgenic littermates were used as controls (WT). Female and male mice were used, and they were equally distributed in each series of experiments. Typization was performed to analyze tissue extracts from tail tips (homogenized in phosphate-buffer saline, freeze/thawed twice, and centrifuged at 23,000× *g* for 15 min at 4 °C) by staining for SOD1 after polyacrylamide gel electrophoresis (10% resolving and 4% stacking) [49,79]. In SOD1^G93A^ mice, death usually occurs between 120 and 140 days. For experimental use, animals were killed at 30, 60, and 90 days of life, corresponding to pre- (30 and 60 days) and early-symptomatic (90 days) stages of the disease. Animals were housed at a constant temperature (22 ± 1 °C) and relative humidity (50%) with a regular 12:12 h light cycle (light 7am and 7pm), throughout the experiments. Food and water were freely available. All experiments were carried out in accordance with the guidelines established by the European Communities Council (Directive 114 2010/63/EU of September 22nd, 2010) and with Italian D.L. n. 26/2014 and were approved by the local Ethical Committee and by the Italian Ministry of Health (Project No. 75f11.2, Authorization No.97/2017-PR). All efforts were made to minimize animal suffering and to use only the number of animals necessary to produce reliable results.

### 4.2. Synaptosomes Purification

Animals were euthanized and the whole spinal cord rapidly removed. Synaptosomes were prepared essentially as described previously [80,81]. The tissue was homogenized in 15 volumes of 0.32 M sucrose, buffered at pH 7.4 with Tris-HCl, and using a glass-Teflon tissue grinder (clearance 0.25 mm). The homogenate was centrifuged (5 min, 1000× *g* at 4 °C) to remove nuclei and debris and the supernatant was harvested and centrifuged at 12,000× *g* for 10 min. The pellet was suspended in Tris-buffered 0.32 M sucrose and gently layered on a discontinuous Percoll^®^ (Sigma-Aldrich, St Louis, MO, USA) gradient (2%, 6%, 10%, and 20% v/v in Tris-buffered 0.32 M sucrose). After centrifugation at 33,500× *g* for 5 min, the layer between 10% and 20% Percoll^®^, corresponding to synaptosomal fraction, was collected and washed by centrifugation at 20,000× *g* for 15 min in physiological medium, having the following compositions (mM): NaCl, 140; KCl, 3; MgSO_4_, 1.2; NaH_2_PO_4_, 1.2; NaHCO_3_, 5; CaCl_2_, 1.2; 4-(2-hydroxyethyl)-1-piperazineethanesulfonic acid (HEPES), 10; glucose, 10; pH 7.4. The synaptosomal pellet was then resuspended in physiological medium for cytosolic calcium concentration ([Ca^2+^]c) determination, release and confocal microscopy experiments, or in lysis buffer for Western blotting. Protein content was measured according to Bradford [82] using bovine serum albumin (BSA; Sigma-Aldrich, St Louis, MO, USA) as standard. All the reagents were of laboratory grade.

### 4.3. Release Experiments

Synaptosomes from 30-, 60-, and 90-day-old WT and SOD1^G93A^ mice were incubated at 37 °C for 15 min in the presence of 0.1 µM [^3^H]d-Aspartate ([^3^H]d-Asp), a non-metabolizable analogue of glutamate used to label the glutamate-releasing pools [83,84]. The synaptosomal suspension was equally distributed on microporous filters placed at the bottom of a set of 24 superfusion chambers maintained at 37 °C (Superfusion System, Ugo Basile, Comerio, Varese, Italy) [85,86]. Superfusion started with standard medium at a rate of 0.5 mL/min and continued for 48 min. After 36 min of superfusion to equilibrate the system, five 3-min samples were collected. (*s*)-3,5-Dihydroxyphenylglycine (3,5-DHPG; 0.03–30 µM) was introduced at the end of the first sample collected (*t* = 39 min) and maintained until the end of the experiment; the other drugs used were introduced at *t* = 30 min. When appropriate, Ca^2+^ was omitted from *t* = 20 min. In some experiments, synaptosomes were incubated for 30 min (15 min before and during [^3^H]d-Asp labelling) in the presence of 100 µM 1,2-bis-(2-aminophenoxy)-ethane-N,N,N0,N0-tetraacetic acid tetra acetoxymethyl ester (BAPTA-AM) or with 0.1 µM bafilomycin A1. Collected samples and superfused synaptosomes were counted for radioactivity. Tritium released in each sample was calculated as fractional rate × 100 (percentage of the total synaptosomal neurotransmitter content at the beginning of the respective collection period). Drug effects were evaluated by calculating the ratio between the efflux in the fourth sample collected, in which the maximum effect of 3,5-DHPG was generally reached, and the efflux in the first sample (basal efflux). This ratio was compared to the corresponding ratio obtained in appropriate controls that were always run in parallel.

### 4.4. Confocal Microscopy

Purified synaptosomes (40 µg protein) from 30-, 60-, and 90-day-old WT and SOD1^G93A^ mice were stratified onto poly-l-lysine pre-treated coverslips and maintained for 45 min at room temperature to allow setting and sticking to the surface. The preparations were fixed with 2% para-formaldehyde (15 min), washed with PBS (3 × 5), and permeabilized (5 min) with 0.05% triton X-100. After washing (3 × 5) with PBS containing 0.5% BSA, the preparations were incubated overnight at 4 °C with the primary antibodies diluted in PBS containing 3% BSA. The following antibodies were used: Guinea pig anti-vesicular Glu transporter 1 (vGluT1, 1:1000, cat n. AB5905; Merk Millipore, Billerica, MA, USA), mouse anti-mGluR1 (1:1000, cat n. 610964; BD Biosciences, NJ, USA), and rabbit anti-mGluR5 (1:500, cat n. ab53090; Abcam, Cambridge, UK). After washing (3 × 5) with PBS containing 0.5% BSA, the preparations were incubated for 45 min with the secondary antibodies. The following antibodies were used: Goat anti-guinea pig Alexa Fluor A488-conjugated (cat n. A11073), donkey anti-mouse Alexa Fluor A647-conjugated (cat n. A31571), and goat anti-rabbit AlexaFluor A555-conjugated (cat n. A21428) (Molecular Probes Europe, Leiden, The Netherlands). Antibodies were diluted 1:2000 in PBS containing 3% albumin. Fluorescence image (512 × 512 × 8 bit) acquisition was performed by a three-channel Leica TCS SP2 laser-scanning confocal microscope, equipped with 458, 476, 488, 514, 543, and 633 nm excitation lines, through a plan-apochromatic oil immersion objective 63× (1.4 NA). Light collection configuration was optimized according to the combination of chosen fluorochromes and sequential channel acquisition was performed to avoid crosstalk. Leica “LAS AF” software package was used for image acquisition. The estimation of co-localized proteins was performed by calculating the co-localization coefficients [87].

### 4.5. Immunoblotting

Spinal cord synaptosomal or total tissue proteins from 30-, 60-, and 90-day-old WT and SOD1^G93A^ mice were separated by means of SDS-polyacrylamide gel electrophoresis; 4% to 20% gradient gels were used. The concentration of proteins in each sample fell in the linear portion of the standard curve. Proteins were transferred to nitrocellulose membranes and electroblotted proteins were monitored using Naphthol blue black staining (Sigma Aldrich, MO, USA). Membranes were saturated in 5% skimmed-milk solution and incubated with the following antibodies: Mouse monoclonal anti-mGluR1 (1:500, cat n. 610964; BD Biosciences, San Jose, CA, USA); rabbit monoclonal anti-mGluR5 (1:500, cat n. ab53090; Abcam, Cambridge, UK); mouse monoclonal anti-glyceraldeide phosphate dehydrogenase, GAPDH (1:10000, cat. N. G8795; Sigma Aldrich, MO, USA); mouse monoclonal anti-β-tubulin III (1:1000; cat. N. T8578; Sigma-Aldrich, MO, USA). After incubation with peroxidase-coupled secondary antibodies, protein bands were detected and analyzed for optical density using an enhanced chemiluminescence substrate (ECL, LiteAblot PLUS, Euroclone, Milan, Italy) and a chemiluminescence system (Alliance 6.7 WL 20M, UVITEC, Cambridge, UK), and UV1D software (UVITEC). Bands of interest were normalized for β-tubulin III or GAPDH levels in the same membrane.

### 4.6. Cytosolic Ca^2+^ Concentration

[Ca^2+^]_C_ was determined in spinal cord synaptosomes from 30-, 60-, and 90-day-old WT and SOD1^G93A^ mice using the fluorescent dye fura 2-AM [79]. Synaptosomes were incubated for 40 min at 37 °C in physiological medium, in the presence of 20 mM CaCl_2_ and 5 mM fura 2-AM dissolved in 0.5% dimethyl sulfoxide (DMSO; Sigma-Aldrich, St Louis, MO, USA). Synaptosomes incubated in the presence of 0.5% DMSO only were used to measure auto-fluorescence. Extra-synaptosomal fura 2-AM was removed by centrifugation; pellets were re-suspended in ice-cold Ca^2+^-free physiological medium, divided into 200-µL aliquots (200 µg protein/sample), and stored on ice until use. All measurements were obtained within 2 h. Synaptosomes were diluted in a final volume of 2 mL of physiological medium containing 1.2 mM CaCl_2_ and equilibrated at 37 °C for 15 min. The measurements were made at 37 °C under continuous stirring using an RF-5301PC dual wavelength spectrofluorophotometer (Shimadzu Corporation, Milan, Italy) by alternating the excitation wavelengths of 340 and 380 nm. Fluorescent emission was monitored at 510 nm. Basal fluorescence was recorded for 1 min, then synaptosomes were exposed to 3,5-DHPG (0.3 or 30 µM) for an additional 8 min. Calibration of the fluorescent signals was performed at the end of each measurement by adding 10 mM ionomycin in the presence of CaCl_2_ to obtain F_max_, followed by 10 mM EDTA (pH 8.0, buffered with 3 mM Tris) to obtain F_min_. After correcting for extracellular dye, [Ca^2+^]_C_ was calculated by the equation of Grynkiewicz et al. [88], using a K_D_ of 224 nM for the Ca^2+^-fura 2-AM complex.

### 4.7. Statistics

Data are expressed as mean ± SEM. The Shapiro–Wilk normality test was performed to assess the normality of the data. Grubbs’ test for outliers was performed. Statistical comparison between two means was performed by two-tailed student’s *t*-test while multiple comparisons were performed by using the analysis of variance (one or two-way ANOVA, as appropriate), followed by the Bonferroni’s post hoc test or Dunnet’s test, and *p*-values lower than 0.05 were considered significant. Analyses were carried out using the SigmaStat software (Systat Software 3.5, Inc., San Jose, CA, USA).
